# 2-(Prop-2-enyloxy)benzamide

**DOI:** 10.1107/S1600536812042250

**Published:** 2012-10-20

**Authors:** Bernhard Bugenhagen, Yosef Al Jasem, Farah Barkhad, Bassam al Hindawi, Thies Thiemann

**Affiliations:** a Institute of Inorganic Chemistry, University of Hamburg, Hamburg, Germany; b Department of Chemical Engineering, UAE University, AL Ain, Abu Dhabi, United Arab Emirates; c Department of Petroleum Engineering, UAE University, AL Ain, Abu Dhabi, United Arab Emirates; d Department of Chemistry, UAE University, AL Ain, Abu Dhabi, United Arab Emirates

## Abstract

In the title mol­ecule, C_10_H_11_NO_2_, the benzene ring forms dihedral angles of 33.15 (2) and 6.20 (2)° with the mean planes of the amide and propen­oxy groups, respectively. The amide –NH_2_ group is oriented toward the propen­oxy substituent and forms a weak intra­molecular N—H⋯O hydrogen bond to the propen­oxy O atom. The conformation of the propen­oxy group at the C*sp* ^2^—C*sp* ^3^ and C*sp* ^3^—O bonds is *synperiplanar* and *anti­periplanar*, respectively. In the crystal, N—H⋯O hydrogen bonds involving the amide groups generate *C*(4) and *R* ^2^_3_(7) motifs that organize the mol­ecules into tapes along the *a-*axis direction. There are C—H⋯π inter­actions between the propen­oxy –CH_2_ group and the aromatic system of neighboring mol­ecules within the tape. The mean planes of the aromatic ring and the propen­oxy group belonging to mol­ecules located on opposite sites of the tape form an angle of 83.16 (2)°.

## Related literature

For crystal structures of similar compounds, see: Al Jasem *et al.* (2012 ▶); Pagola & Stephens (2009 ▶); Johnstone *et al.* (2010 ▶); Pertlik (1990) ▶; Sasada *et al.* (1964 ▶). For uses of 2-alk­oxy­benzamides, see: van de Waterbeemd & Testa (1983 ▶); Kusunoki & Harada (1984 ▶). For the preparation of a related 2-alk­oxy­benzamide, see: Al Jasem *et al.* (2012 ▶).
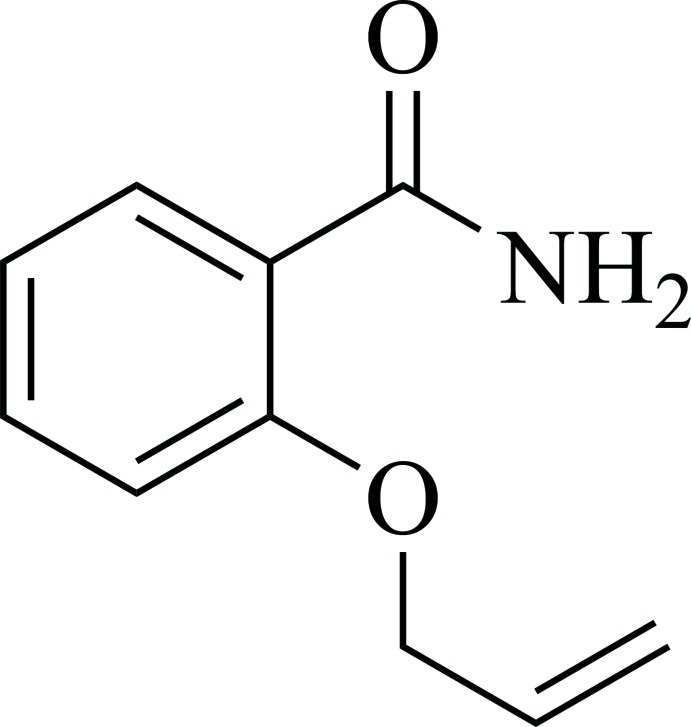


## Experimental

### 

#### Crystal data

C_10_H_11_NO_2_
*M* *_r_* = 177.20
Orthorhombic, 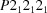

*a* = 5.08891 (17) Å
*b* = 11.2542 (4) Å
*c* = 15.8802 (6) Å
*V* = 909.48 (5) Å^3^
*Z* = 4
Cu *K*α radiation
μ = 0.74 mm^−1^
*T* = 100 K
0.30 × 0.09 × 0.08 mm

#### Data collection

Agilent SuperNova Atlas diffractometer
Absorption correction: Gaussian (*CrysAlis PRO*; Agilent, 2012 ▶) *T*_min_ = 0.862, *T*_max_ = 0.951
4718 measured reflections
1079 independent reflections
1016 reflections with *I* > 2σ(*I*)
*R*_int_ = 0.025

#### Refinement

*R*[*F* ^2^ > 2σ(*F* ^2^)] = 0.033
*wR*(*F* ^2^) = 0.087
*S* = 1.03
1079 reflections
126 parameters
H atoms treated by a mixture of independent and constrained refinement
Δρ_max_ = 0.17 e Å^−3^
Δρ_min_ = −0.18 e Å^−3^

### 

Data collection: *CrysAlis PRO* (Agilent, 2012 ▶); cell refinement: *CrysAlis PRO*; data reduction: *CrysAlis PRO*; program(s) used to solve structure: *SHELXS97* (Sheldrick, 2008 ▶); program(s) used to refine structure: *SHELXL97* (Sheldrick, 2008 ▶) within *OLEX2* (Dolomanov *et al.*, 2009 ▶); molecular graphics: *PLATON* (Spek, 2009 ▶); *Mercury* (Macrae *et al.*, 2008 ▶); software used to prepare material for publication: *SHELXL97*, *PLATON*.

## Supplementary Material

Crystal structure: contains datablock(s) global, I. DOI: 10.1107/S1600536812042250/gk2521sup1.cif

Structure factors: contains datablock(s) I. DOI: 10.1107/S1600536812042250/gk2521Isup2.hkl

Supplementary material file. DOI: 10.1107/S1600536812042250/gk2521Isup3.cml

Additional supplementary materials:  crystallographic information; 3D view; checkCIF report

## Figures and Tables

**Table 1:** Hydrogen-bond geometry (Å, °) *Cg* is the centroid of the C1–C6 ring.

*D*—H⋯*A*	*D*—H	H⋯*A*	*D*⋯*A*	*D*—H⋯*A*
N1—H1*A*⋯O1^i^	0.90 (2)	2.01 (2)	2.905 (2)	178 (17)
N1—H1*B*⋯O1^ii^	0.89 (3)	2.12 (3)	2.863 (2)	140 (2)
N1—H1*B*⋯O2	0.89 (3)	2.31 (2)	2.754 (2)	110.8 (18)
C8—H8*B*⋯*Cg* ^ii^	0.99	2.68	3.461 (2)	137

Symmetry codes: (i) 
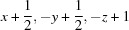
; (ii) 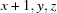.

**Fig. 1.**
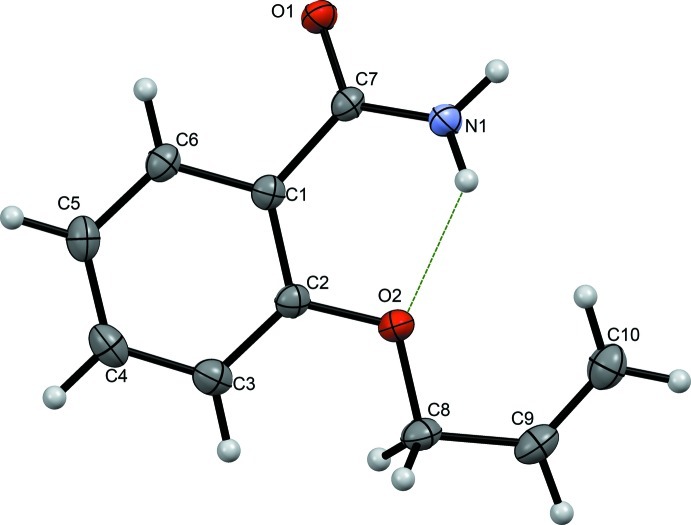
A view of the title compound molecule with the atom-numbering scheme and the intramolecular interaction within the molecule. Displacement ellipsoids are shown at the 50% probability level.

**Fig. 2.**
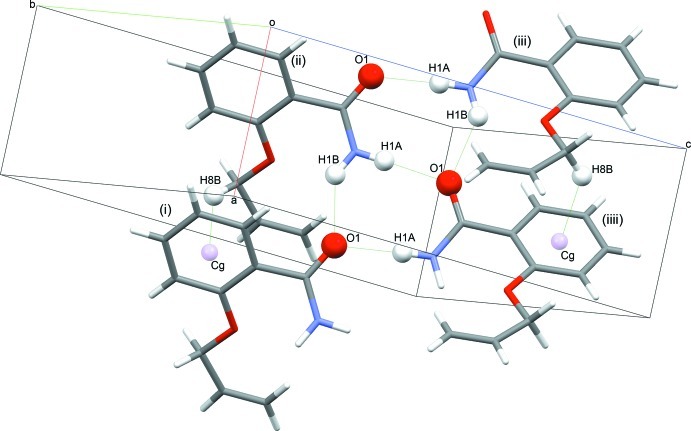
Intermolecular attractions between molecules of the title compound. [Symmetry codes: i: 1+x,y,z; ii: x,y,z; iii: -1/2 + x,1/2 - y,1 - z; iiii: 1/2 + x, 1/2 - y,1 - z]

**Fig. 3.**
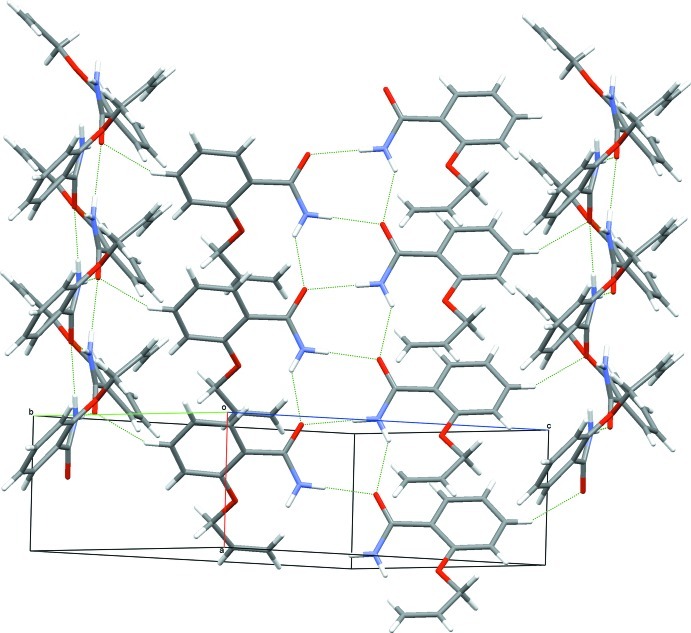
The crystal packing diagram showing the C—H···O intermolecular interactions between tapes formed via amide group interactions.

## supplementary crystallographic information

### Comment

In 2-propenoxybenzamide (2-allyloxybenzamide) (Figure 1), the O1—C7—C1—C6 torsion angle characterizing the twist of the benzene ring relative to the amide group is -30.3 (2)° and the corresponding C8—O2—C2—C3 torsion angle for the propoxy group is 5.9 (2)°. There is an intramolecular N1—H1B···O2 bond within each molecule (Table 1). When compared to the structurally comparable 2-propoxybenzamide (Al Jasem *et al.*, 2012), the torsion angle O1—C7—C1—C6 is much larger in the title compound. The amide groups generate C(4) and R^2^_3_(7) hydrogen-bond motifs that organize the molecules into tapes along the *a* axis. The title compound exhibits a C10—H10A···O2 and a C8—H8··· π (Table 1) close contact, absent in 2-propoxybenzamide (Figure 2). The C4—H4···O1 intermolecular interaction in 2-propenoxybenzamide links the neighboring tapes of molecules along the *a* axis with each other (Figure 3). However, in 2-propoxybenzamide, where also a C–H···O intermolecular interaction is found, the interaction proceeds from the carbon *ortho* to the propoxy group, while in the present case, it proceeds from the carbon *meta* to the propenoxy group. As a result of more close intermolecular contacts in 2-propenoxybenzamide as compared to 2-propoxybenzamide, the difference in the packing between the two compounds is large. The main difference is that while in the 2-propoxybenzamide molecules are arranged into pairs by close contacts, where the pairs in one layer are not associated through close contacts, in the title compound all neighboring molecules form close contacts to each other. Nevertheless, both compounds exhibit particular molecular tapes, each compound with two different directions of tape propagation. In the title compound, the average plane (0 1 - 1) of a tape propagation has an angle of 68.78 (2)° with the corresponding plane (0 1 1) of the neighboring tape propagation. Due to the large dihedral angle between the benzene ring and the amide group in 2-propenoxybenzamide, the average plane (-1 2 2) of the benzene ring and the propenoxy group of a molecule in one stack makes an angle of 83.16 (2)° with the corresponding plane (1 2 2) of a molecule in the opposing motif within one tape.

### Experimental

To powdered KOH (1.12 g, 20.0 mmol) in DMSO (18 ml) was added salicylamide (2.74 g, 20.0 mmol), and the resulting mixture was stirred for 10 min. at rt. Thereafter, *n*-propenyl bromide (4.2 g, mmol, 34.7 mmol) was added dropwise. The solution was stirred for 12 h at rt. Then, it was poured into water (200 ml) and extracted with chloroform (3 *x* 75 ml). The organic phase was dried over anhydrous MgSO_4_, concentrated *in vacuo*, and the residue was subjected to column chromatography on silica gel (CHCl_3_/*M^t^*BE/hexane *v/v/v* 1:1:1) to give 2-propenoxybenzamide (2.76 g, 78%) as colorless crystals (m.p. 377 K). The crystal was grown from CHCl_3_/ *M^t^*BE/hexane (*v/v/v* 1:1:1).IR (KBr) ν_max_ 3406, 3190, 1631, 1600, 1399, 1243, 996, 921, 757, 643, 627 cm^-1^; δ_H_ (400 MHz, CDCl_3_) 4.67 (2*H*, d, ^3^*J* = 5.6 Hz), 5.36 (1*H*, dd, ^3^*J* = 10.4 Hz, ^2^*J* = 1.2 Hz), 5.44 (1*H*, dd, ^3^*J* = 17.2 Hz, ^2^*J* = 1.2 Hz), 6.03 – 6.13 (1*H*, dt, ^3^*J* = 17.2 Hz, ^3^*J* = 10.4 Hz, ^3^*J* = 5.6 Hz), 6.25 (1*H*, bs, NH), 6.96 (1*H*, d, ^3^*J* = 8.0 Hz), 7.07 (1*H*, dd, ^3^*J* = 8.0 Hz, ^3^*J* = 8.0 Hz), 7.80 (1*H*, bs, NH), 8.20 (1*H*, dd, ^3^*J* = 8.0 Hz, ^4^*J* = 1.6 Hz); δ_C_ (100.5 MHz, CDCl_3_) 69.9, 112.6, 119.4, 121.1, 121.4, 132.0, 132.6, 133.3, 156.9, 167.2.

### Refinement

All carbon-bound hydrogen atoms were placed in calculated positions with C—H

distances of 0.95 - 0.99 Å and refined as riding with *U*_iso_(H)

=*xU*_eq_(C), where *x* = 1.5 for methyl and *x* = 1.2 for all other H-atoms.

The N-bound H atom positions were determined from difference electron

density map and refined freely. In the absence of significant anomalous

scattering effects Friedel pairs have been merged.

### Crystal data

**Table:**

C_10_H_11_NO_2_	*D*_x_ = 1.294 Mg m^−^^3^
*M**_r_* = 177.20	Melting point: 377 K
Orthorhombic, *P*2_1_2_1_2_1_	Cu *K*α radiation, λ = 1.5418 Å
*a* = 5.08891 (17) Å	Cell parameters from 2824 reflections
*b* = 11.2542 (4) Å	θ = 3.9–72.6°
*c* = 15.8802 (6) Å	µ = 0.74 mm^−^^1^
*V* = 909.48 (5) Å^3^	*T* = 100 K
*Z* = 4	Needle, colourless
*F*(000) = 376	0.30 × 0.09 × 0.08 mm

### Data collection

**Table:**

Agilent SuperNova Atlas diffractometer	1079 independent reflections
Radiation source: SuperNova (Cu) X-ray Source	1016 reflections with *I* > 2σ(*I*)
Mirror monochromator	*R*_int_ = 0.025
Detector resolution: 10.4127 pixels mm^-1^	θ_max_ = 72.7°, θ_min_ = 4.8°
ω scans	*h* = −6→3
Absorption correction: gaussian (*CrysAlis PRO*; Agilent, 2012)	*k* = −12→13
*T*_min_ = 0.862, *T*_max_ = 0.951	*l* = −19→19
4718 measured reflections	

### Refinement

**Table:**

Refinement on *F*^2^	Primary atom site location: structure-invariant direct methods
Least-squares matrix: full	Secondary atom site location: difference Fourier map
*R*[*F*^2^ > 2σ(*F*^2^)] = 0.033	Hydrogen site location: inferred from neighbouring sites
*wR*(*F*^2^) = 0.087	H atoms treated by a mixture of independent and constrained refinement
*S* = 1.03	*w* = 1/[σ^2^(*F*_o_^2^) + (0.0609*P*)^2^ + 0.1267*P*] where *P* = (*F*_o_^2^ + 2*F*_c_^2^)/3
1079 reflections	(Δ/σ)_max_ < 0.001
126 parameters	Δρ_max_ = 0.17 e Å^−^^3^
0 restraints	Δρ_min_ = −0.18 e Å^−^^3^

### Special details

**Table:**

Experimental. Numerical absorption correction based on gaussian integration over a multifaceted crystal model
Geometry. All e.s.d.'s (except the e.s.d. in the dihedral angle between two l.s. planes) are estimated using the full covariance matrix. The cell e.s.d.'s are taken into account individually in the estimation of e.s.d.'s in distances, angles and torsion angles; correlations between e.s.d.'s in cell parameters are only used when they are defined by crystal symmetry. An approximate (isotropic) treatment of cell e.s.d.'s is used for estimating e.s.d.'s involving l.s. planes.
Refinement. Refinement of *F*^2^ against ALL reflections. The weighted *R*-factor *wR* and goodness of fit *S* are based on *F*^2^, conventional *R*-factors *R* are based on *F*, with *F* set to zero for negative *F*^2^. The threshold expression of *F*^2^ > σ(*F*^2^) is used only for calculating *R*-factors(gt) *etc*. and is not relevant to the choice of reflections for refinement. *R*-factors based on *F*^2^ are statistically about twice as large as those based on *F*, and *R*- factors based on ALL data will be even larger.

### Fractional atomic coordinates and isotropic or equivalent isotropic displacement parameters (Å^2^)

**Table:**

	*x*	*y*	*z*	*U*_iso_*/*U*_eq_	
C1	0.2397 (4)	0.43317 (15)	0.31664 (10)	0.0181 (4)	
C10	0.9389 (4)	0.59453 (19)	0.49447 (12)	0.0289 (4)	
C2	0.4129 (3)	0.52840 (15)	0.30222 (11)	0.0188 (4)	
C3	0.3949 (4)	0.59415 (17)	0.22777 (12)	0.0238 (4)	
C4	0.2055 (4)	0.56545 (18)	0.16826 (11)	0.0262 (4)	
C5	0.0314 (4)	0.47271 (17)	0.18190 (11)	0.0244 (4)	
C6	0.0478 (4)	0.40808 (16)	0.25658 (11)	0.0208 (4)	
C7	0.2401 (3)	0.35748 (15)	0.39466 (11)	0.0181 (4)	
C8	0.7520 (4)	0.65550 (15)	0.35506 (12)	0.0231 (4)	
C9	0.9255 (4)	0.66806 (17)	0.43007 (12)	0.0268 (4)	
H10A	0.8310	0.5257	0.4958	0.035*	
H10B	1.0565	0.6104	0.5396	0.035*	
H1A	0.484 (5)	0.288 (2)	0.4772 (13)	0.028 (6)*	
H1B	0.623 (5)	0.358 (2)	0.4113 (16)	0.040 (7)*	
H3	0.5120	0.6584	0.2179	0.029*	
H4	0.1950	0.6099	0.1175	0.031*	
H5	−0.0975	0.4534	0.1408	0.029*	
H6	−0.0741	0.3458	0.2667	0.025*	
H8A	0.6389	0.7267	0.3496	0.028*	
H8B	0.8604	0.6488	0.3035	0.028*	
H9	1.0373	0.7356	0.4316	0.032*	
N1	0.4686 (3)	0.33298 (15)	0.43084 (10)	0.0218 (3)	
O1	0.0291 (2)	0.31561 (12)	0.42082 (8)	0.0224 (3)	
O2	0.5922 (2)	0.55184 (11)	0.36411 (7)	0.0217 (3)	

### Atomic displacement parameters (Å^2^)

**Table:**

	*U*^11^	*U*^22^	*U*^33^	*U*^12^	*U*^13^	*U*^23^
C1	0.0162 (8)	0.0185 (8)	0.0197 (8)	0.0023 (7)	0.0017 (7)	−0.0006 (6)
C2	0.0149 (8)	0.0194 (8)	0.0221 (8)	0.0015 (7)	0.0019 (7)	0.0001 (7)
C3	0.0226 (9)	0.0230 (8)	0.0259 (9)	0.0029 (8)	0.0040 (7)	0.0037 (7)
C4	0.0309 (10)	0.0275 (10)	0.0202 (8)	0.0076 (9)	0.0018 (8)	0.0044 (7)
C5	0.0245 (9)	0.0280 (9)	0.0207 (8)	0.0049 (8)	−0.0038 (7)	−0.0032 (7)
C6	0.0180 (8)	0.0205 (8)	0.0239 (8)	0.0014 (7)	−0.0004 (8)	−0.0028 (7)
C7	0.0159 (8)	0.0173 (8)	0.0210 (8)	0.0005 (7)	0.0006 (7)	−0.0019 (6)
C8	0.0213 (9)	0.0177 (8)	0.0304 (9)	−0.0038 (8)	0.0011 (8)	0.0008 (7)
C9	0.0211 (9)	0.0240 (9)	0.0353 (10)	−0.0040 (8)	0.0013 (8)	−0.0066 (8)
C10	0.0270 (10)	0.0315 (9)	0.0283 (9)	0.0002 (9)	−0.0022 (9)	−0.0070 (8)
N1	0.0153 (7)	0.0260 (8)	0.0240 (7)	−0.0007 (6)	0.0000 (6)	0.0071 (6)
O1	0.0153 (6)	0.0243 (6)	0.0276 (6)	−0.0017 (5)	0.0006 (5)	0.0053 (5)
O2	0.0195 (6)	0.0210 (6)	0.0246 (6)	−0.0045 (5)	−0.0015 (5)	0.0033 (5)

### Geometric parameters (Å, º)

**Table:**

C1—C2	1.406 (2)	C7—N1	1.326 (2)
C1—C6	1.394 (2)	C7—O1	1.244 (2)
C1—C7	1.504 (2)	C8—H8A	0.9900
C2—C3	1.398 (2)	C8—H8B	0.9900
C2—O2	1.367 (2)	C8—C9	1.489 (3)
C3—H3	0.9500	C8—O2	1.429 (2)
C3—C4	1.388 (3)	C9—H9	0.9500
C4—H4	0.9500	C9—C10	1.317 (3)
C4—C5	1.386 (3)	C10—H10A	0.9500
C5—H5	0.9500	C10—H10B	0.9500
C5—C6	1.394 (2)	N1—H1A	0.90 (2)
C6—H6	0.9500	N1—H1B	0.89 (3)
			
C1—C6—H6	119.4	C7—N1—H1A	123.2 (16)
C10—C9—C8	126.30 (18)	C7—N1—H1B	124.0 (16)
C10—C9—H9	116.9	C8—C9—H9	116.9
C2—C1—C7	124.39 (15)	C9—C10—H10A	120.0
C2—C3—H3	120.1	C9—C10—H10B	120.0
C2—O2—C8	117.72 (13)	C9—C8—H8A	109.8
C3—C2—C1	120.00 (16)	C9—C8—H8B	109.8
C3—C4—H4	119.6	H10A—C10—H10B	120.0
C4—C3—C2	119.89 (18)	H1A—N1—H1B	113 (2)
C4—C3—H3	120.1	H8A—C8—H8B	108.2
C4—C5—H5	120.4	N1—C7—C1	118.44 (16)
C4—C5—C6	119.20 (17)	O1—C7—C1	119.23 (15)
C5—C4—C3	120.84 (17)	O1—C7—N1	122.25 (16)
C5—C4—H4	119.6	O2—C2—C1	116.64 (14)
C5—C6—C1	121.22 (17)	O2—C2—C3	123.36 (16)
C5—C6—H6	119.4	O2—C8—H8A	109.8
C6—C1—C2	118.81 (15)	O2—C8—H8B	109.8
C6—C1—C7	116.76 (16)	O2—C8—C9	109.54 (15)
C6—C5—H5	120.4		

### Hydrogen-bond geometry (Å, º)

Cg is the centroid of the C1–C6 ring.

**Table:**

*D*—H···*A*	*D*—H	H···*A*	*D*···*A*	*D*—H···*A*
N1—H1*A*···O1^i^	0.90 (2)	2.01 (2)	2.905 (2)	178 (17)
N1—H1*B*···O1^ii^	0.89 (3)	2.12 (3)	2.863 (2)	140 (2)
N1—H1*B*···O2	0.89 (3)	2.31 (2)	2.754 (2)	110.8 (18)
C8—H8*B*···*Cg*^ii^	0.99	2.68	3.461 (2)	137

Symmetry codes: (i) *x*+1/2, −*y*+1/2, −*z*+1; (ii) *x*+1, *y*, *z*.
